# Distinct Neural Correlates of Linguistic and Non-Linguistic Demand

**DOI:** 10.1162/nol_a_00031

**Published:** 2021-03-29

**Authors:** Ian A. Quillen, Melodie Yen, Stephen M. Wilson

**Affiliations:** Department of Hearing and Speech Sciences, Vanderbilt University Medical Center, Nashville, TN, USA

**Keywords:** linguistic demand, task difficulty, accuracy, reaction time, multiple demand network, aphasia

## Abstract

In this study, we investigated how the brain responds to task difficulty in linguistic and non-linguistic contexts. This is important for the interpretation of functional imaging studies of neuroplasticity in post-stroke aphasia, because of the inherent difficulty of matching or controlling task difficulty in studies with neurological populations. Twenty neurologically normal individuals were scanned with fMRI as they performed a linguistic task and a non-linguistic task, each of which had two levels of difficulty. Critically, the tasks were matched across domains (linguistic, non-linguistic) for accuracy and reaction time, such that the differences between the easy and difficult conditions were equivalent across domains. We found that non-linguistic demand modulated the same set of multiple demand (MD) regions that have been identified in many prior studies. In contrast, linguistic demand modulated MD regions to a much lesser extent, especially nodes belonging to the dorsal attention network. Linguistic demand modulated a subset of language regions, with the left inferior frontal gyrus most strongly modulated. The right hemisphere region homotopic to Broca’s area was also modulated by linguistic but not non-linguistic demand. When linguistic demand was mapped relative to non-linguistic demand, we also observed domain by difficulty interactions in temporal language regions as well as a widespread bilateral semantic network. In sum, linguistic and non-linguistic demand have strikingly different neural correlates. These findings can be used to better interpret studies of patients recovering from aphasia. Some reported activations in these studies may reflect task performance differences, while others can be more confidently attributed to neuroplasticity.

## INTRODUCTION

How does the brain respond to task difficulty in linguistic and non-linguistic contexts? Our motivation for addressing this question is that it bears on the interpretation of functional imaging studies of neuroplasticity in post-stroke aphasia. Individuals with aphasia, by their nature, find language tasks more difficult than do neurologically normal control participants. Moreover, as patients recover over time, language tasks generally become easier. These facts imply that comparisons between patients and controls, and longitudinal analyses as patients recover, are confounded by task difficulty. Therefore, when activation differences are observed, it is difficult to determine whether they reflect functional reorganization or effects of task difficulty (Binder et al., 2005; Fridriksson & Morrow, 2005; Geranmayeh et al., 2014; Price et al., 2006). Attempts have been made to manipulate and match task difficulty between patients and controls (Brownsett et al., 2014; Raboyeau et al., 2008; Sharp et al., 2004; Sharp et al., 2010; Wilson et al., 2018; Wilson et al., 2019), but precise matching has proven difficult to achieve.

The brain regions that are modulated by task difficulty are generally remarkably consistent across tasks. A bilateral network including the inferior frontal junction, anterior insula, pre-supplementary motor area (SMA), anterior-mid cingulate, and intraparietal sulcus has been described as constituting a “multiple-demand” (MD) network, supporting cognitive flexibility in many contexts (Duncan & Owen, 2000; Fox et al., 2005). A compelling demonstration of the generality of this network came from a study in which comparisons between difficult and easy conditions of seven diverse cognitive tasks yielded similar patterns of fronto-parietal activation (Fedorenko et al., 2013).

Based on these findings, it could be speculated that the additional task difficulty that individuals with aphasia experience when performing language tasks would yield increased activity in MD regions (Geranmayeh et al., 2014). However, it is not clear that linguistic demand is analogous to other types of cognitive demand. While two of the seven tasks investigated by Fedorenko et al. (2013) involved linguistic stimuli, both of these were verbal working memory tasks. Many previous studies have manipulated linguistic factors such as syntactic complexity, ambiguity, word frequency, or difficulty of semantic decisions. Some studies have reported that these manipulations modulated likely MD regions (Binder et al., 2004; Binder et al., 2005; Blumstein et al., 2005; Eckert et al., 2009; Erb et al., 2013; Ihnen et al., 2015; Mollica et al., 2020; Noppeney & Price, 2004; Piai et al., 2013; Sabsevitz et al., 2005; Vaden et al., 2013; Wilson et al., 2016); while others have reported modulation of likely left hemisphere language regions (Binder et al., 2005; Bornkessel et al., 2005; Graves et al., 2007; Graves et al., 2010; Just et al., 1996; Makuuchi et al., 2009; Mason et al., 2003; Obleser et al., 2011; Rodd et al., 2005; Roskies et al., 2001; Sabsevitz et al., 2005; Stromswold et al., 1996; Thompson et al., 2007; Wilson et al., 2016). Often it is difficult to determine whether language or MD regions are involved, given the close proximity of some of these regions (Fedorenko et al., 2012), and the fact that most studies have not explicitly assessed both possibilities. Only a few studies have manipulated both linguistic and non-linguistic demand, with modulation in common most often observed in the anterior insula and/or anterior cingulate (Eckert et al., 2009; Erb et al., 2013; Piai et al., 2013). No studies, to our knowledge, have matched task structure and behavioral factors across linguistic and non-linguistic tasks so as to permit direct statistical comparisons.

The goal of the present study is to directly compare the brain regions that are modulated by linguistic demand and non-linguistic demand. This entails matching task structure across domains and ensuring that the difference in difficulty between easy and difficult conditions is precisely matched across domains in terms of accuracy and reaction time. A clear picture of similarities and differences between the neural correlates of linguistic demand and non-linguistic demand may facilitate the interpretation of studies of neuroplasticity in aphasia, in which task difficulty is so notoriously hard to control.

## METHODS

### Participants

Twenty neurologically normal individuals (age 26.6 ± 6.1 (*SD*) years, range 18–40 years; 3 male, 17 female; 17 right-handed, 3 left-handed; all native speakers of English; education 15.7 ± 1.5 years, range 12–18 years) were successfully scanned with fMRI. Participants were recruited by word of mouth from Vanderbilt University Medical Center community in Nashville, Tennessee. All participants, including the three left-handed participants, were left-lateralized for language, as revealed by contrasts of semantic and perceptual conditions.

Prior to running the imaging study, a separate group of seven participants (age 26.7 ± 3.4 years, range 23–30 years; 2 male, 5 female; all right-handed; all native speakers of English; education 16.9 ± 0.9 years, range 16–18 years) took part in behavioral studies for optimizing the experimental design. These participants were recruited similarly; none of them were scanned.

All participants gave written informed consent and were compensated for their time. The study was approved by the institutional review board at Vanderbilt University Medical Center.

### Experimental Design

**Figure 1.**
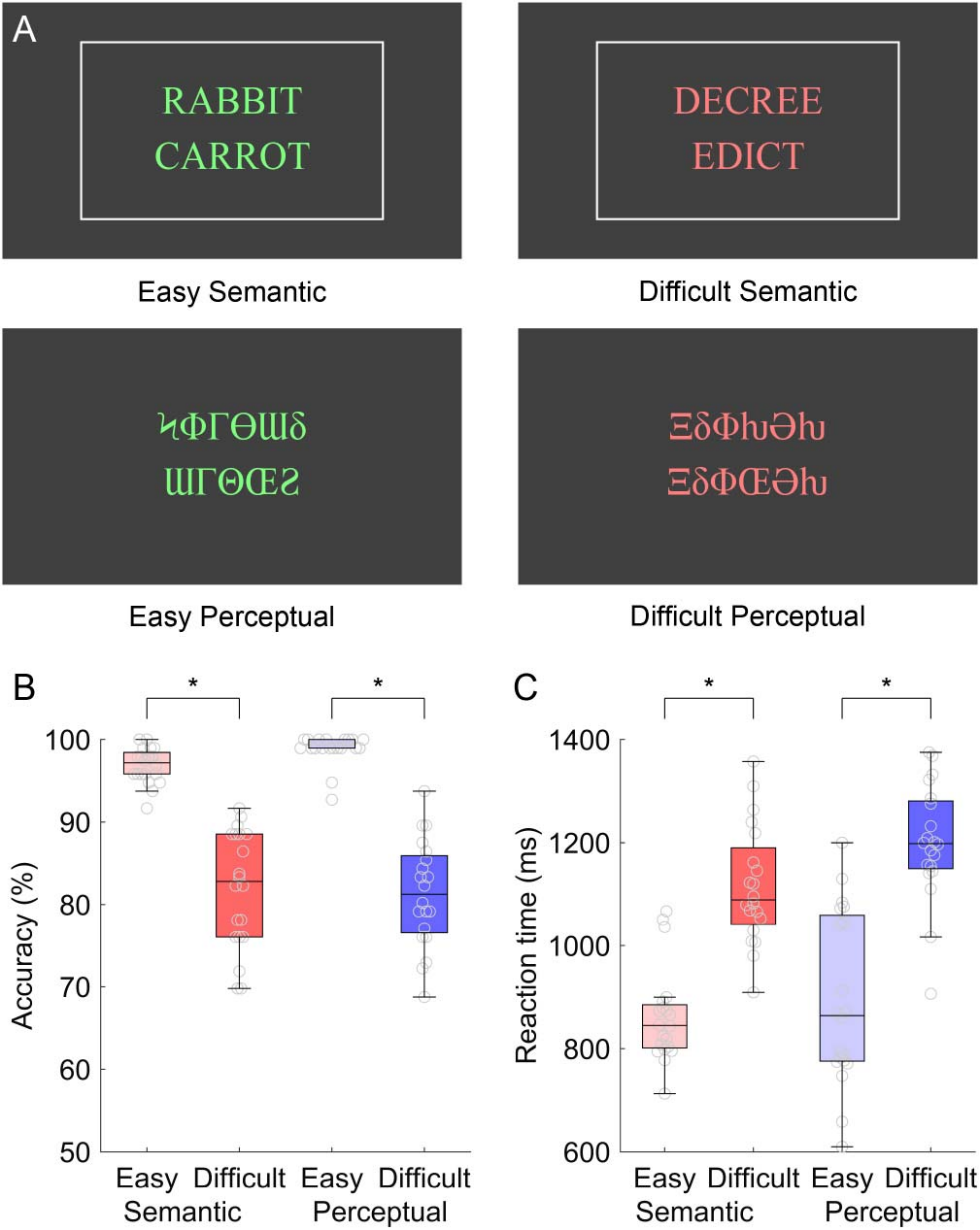
Experimental design and behavioral data. (A) Example items from the four conditions. Matching semantic items are shown, as indicated by the white box that appeared when participants pressed the “match” button. Mismatching perceptual items are shown. Note that items were presented in green or red to cue participants as to the difficulty of the current condition. (B) Accuracy by condition. (C) Reaction time by condition.

In the fMRI study, five conditions were presented in a block design: (1) Semantic Easy; (2) Semantic Difficult; (3) Perceptual Easy; (4) Perceptual Difficult; (5) Rest (Figure 1). All blocks were 16 s in duration, and each block (except for the rest condition) consisted of eight stimuli, which were presented every 2 s. Each run consisted of six blocks per condition in pseudorandom order, for a total of 30 blocks (i.e., exactly 8 min).

Each participant was first trained on the task with untimed presentation of example items from each condition, and specific instructions as described in detail below. Then, they performed one complete practice run prior to entering the scanner, so that they would be familiarized with the four active conditions and would settle on strategies for each condition. Finally, they performed two runs in the scanner while echo-planar images were acquired.

In the four active conditions, each trial consisted of a pair of words or a pair of symbol strings that were visually presented one above the other in the center of the screen. Participants were instructed to press a button with a finger of their left hand if the words “go together” or if the symbol strings “are identical,” and to do nothing otherwise. If they pressed the button, a box appeared around the words or symbols to acknowledge the button press, but no feedback was provided as to whether the response was correct. The number of matching items varied pseudorandomly by block and ranged from two out of eight (25%) to six out of eight (75%). Responses were recorded between 300 ms and 1,900 ms, but the words or symbol strings disappeared after 1,300 ms. Participants were instructed to respond as quickly as possible, but were informed that responses after the stimuli had disappeared would still count.

In the Semantic Easy condition, half of the word pairs were semantically related, and half were not. The words were relatively high frequency, concrete, and acquired early (Table 1), and the semantic relationships between the matching word pairs were chosen to be relatively transparent. The words were presented in green text so that participants knew when they were performing the easy condition. They were instructed: “Green words are easy. Matches will be obvious: the words will clearly go together. You should be able to respond quickly.” The 96 in-scanner word pairs and 48 practice word pairs for this condition were selected from the easier items of the larger stimulus set described by Wilson et al. (2018).

**Table 1.** Characteristics of the stimuli

Condition	Length	Frequency	Age of acquisition	Concreteness	Match example	Mismatch example
Semantic Easy	11.0 ± 0.8	7.51 ± 0.89	4.62 ± 0.88	558 ± 60	RABBIT	TOMATO
					CARROT	BEACH
Semantic Difficult Sub-level 1	11.0 ± 0.8	5.09 ± 0.94	8.61 ± 0.98	456 ± 87	SENSE	ERRAND
					LOGIC	KERNEL
Semantic Difficult Sub-level 2	11.0 ± 0.8	4.50 ± 0.89	9.63 ± 1.00	438 ± 87	SOAR	WHIFF
					FLUTTER	OUTCOME
Semantic Difficult Sub-level 3	11.0 ± 0.8	3.54 ± 0.88	11.49 ± 1.37	400 ± 84	DECREE	KALE
					EDICT	INCLINE
Perceptual Easy	11.0 ± 0.7	—	—	—	ƟƕΨΓƜ	ϞΦΓƟƜδ
					ƟƕΨΓƜ	ƜΓΘŒƧ
Perceptual Difficult	11.0 ± 1.0	—	—	—	ϞƩЖЖΦ	ΞδΦƕƏƕ
					ϞƩЖЖΦ	ΞδΦŒƏƕ

*Note*. Length is the total number of letters summed across both items in each pair; Frequency is the average log lemma frequency across each pair, based on the American National Corpus (Reppen et al., 2005); Age of acquisition is the average across each pair in years from Kuperman et al. (2012); Concreteness is the average across each pair obtained from the MRC database (Coltheart, 1981).

The Semantic Difficult condition was the same as the Semantic Easy condition except that the words were relatively low frequency, abstract, and acquired later (Table 1), and the semantic relationships between the matching word pairs were chosen to be relatively opaque. In this condition, the words were presented in red text so that participants knew when they were performing the difficult condition. They were instructed: “Red words are difficult. Matches may be more subtle: the relationship between the words may be less obvious. Respond as quickly as you can, but take the time you need. If you need to respond after the words have disappeared from the screen, that is fine.” Although 96 in-scanner word pairs and 48 practice word pairs were needed for this condition, three times that many were selected from the more difficult items of the larger stimulus set described by Wilson et al. (2018), such that there were three sub-levels of difficulty among the difficult items. This was done to facilitate matching of accuracy between conditions. During the experiment, items were selected from the three sub-levels depending on the participant’s current relative accuracies on the Semantic Difficult and Perceptual Difficult conditions. If semantic accuracy exceeded perceptual accuracy by more than 5%, then items from the most difficult sub-level were presented. If semantic accuracy was more than 5% below perceptual accuracy, then items from the least difficult sub-level were presented. Otherwise, items from the middle sub-level were presented.

In the Perceptual Easy condition, the differences between matching and mismatching symbol strings were readily apparent (Table 1). Mismatching strings differed in every symbol and one string was always five symbols long while the other was six symbols long. Matching strings were identical and were five or six symbols long. The symbols were presented in green so that participants knew when they were performing the easy condition. They were instructed: “Green symbols are easy. If they mismatch, they will be very different. You should be able to respond quickly.”

The Perceptual Difficult condition was the same as the Perceptual Easy condition, except that pairs of mismatching strings always had the same number of symbols as each other (either 5 or 6) and only one symbol differed between the two (Table 1). In this condition, the symbols were presented in red so that participants knew when they were performing the difficult condition. They were instructed: “Red symbols are difficult. If they mismatch, only one of the symbols will be different. Respond as quickly as you can, but take the time you need. If you need to respond after the symbols have disappeared from the screen, that is fine.”

Note that the specificity of the instructions for each of the four conditions and the complete 8-min practice run prior to scanning were designed to maximize homogeneity of processing strategies across participants. Also, it was important that participants knew when they were performing easy or hard conditions. Without explicit cues to indicate easy conditions, participants might seek subtle semantic relationships between words in Semantic Easy mismatching items, and would have to scour Perceptual Easy matching items for potentially mismatching symbols, which would result in both easy conditions being more demanding than intended.

### Norming Experiment

To compare the neural correlates of linguistic and non-linguistic demand, we needed differences in accuracy and reaction time to be matched across the linguistic and non-linguistic domains. To achieve this, we ran seven participants on preliminary versions of the experiment without scanning them. Based on these participants’ behavioral data, we iteratively adjusted several aspects of the experimental design until we arrived at the design described above, which seemed likely to yield performance that would be balanced as required in the subsequent imaging study. Specifically, (1) we adjusted the degree of mismatch of the Perceptual Easy and Perceptual Difficult mismatch trials; (2) we adjusted the length of time that the stimuli were shown before being removed from the screen; (3) we introduced color cues to condition difficulty; (4) we tweaked the instructions to encourage quick responses on easy conditions; (5) we made the Semantic Difficult condition adaptive to participant performance; and (6) we decided to have participants perform a complete run before scanning so that they would become familiar with the different strategies entailed by the four conditions.

### Neuroimaging

Participants were scanned on a Philips Achieva 3 Tesla scanner with a 32-channel head coil at the Vanderbilt University Institute of Imaging Science. Visual stimuli were projected onto a screen at the end of the bore, which participants viewed through a mirror mounted to the head coil. T2*-weighted blood oxygen level-dependent (BOLD) echo planar images were collected with the following parameters: 240 volumes + 4 initial volumes discarded; 35 axial slices in interleaved order; slice thickness = 3.0 mm with 0.5 mm gap; field of view = 220 × 220 mm; matrix = 96 × 96; repetition time (TR) = 2,000 ms; echo time (TE) = 30 ms; flip angle = 75°; SENSE factor = 2; voxel size = 2.3 × 2.3 × 3.5 mm. T1-weighted structural images and coplanar T2-weighted images were also acquired.

### Behavioral Data Analysis

The behavioral data were analyzed with repeated measures ANOVAs in JMP version 12.0.1 (SAS Institute). Reaction times from all trials with button presses (i.e., hits and false alarms) were included in the analyses. Within each condition for each participant, reaction times for each trial were clipped at 2.5 standard deviations from the mean for that condition and that participant.

### Neuroimaging Data Analysis

The functional imaging data were first preprocessed with tools from AFNI (Cox, 1996). Head motion was corrected, with six translation and rotation parameters saved for use as covariates. Next, the data were detrended with a Legendre polynomial of degree 2, and smoothed with a Gaussian kernel (FWHM = 6 mm). Then, independent component analysis was performed using the FSL tool *melodic* (Beckmann & Smith, 2004). Noise components were manually identified with reference to the criteria of Kelly et al. (2010) and removed using *fsl_regfilt*.

First level models were fit for each of the two functional runs using boxcar models of each active condition convolved with a hemodynamic response function based on the difference of two gamma density functions (time to first peak = 5.4 s; FWHM = 5.2 s; time to second peak = 15 s; FWHM = 10 s; coefficient of second gamma density = 0.09) with the program *fmrilm* from the FMRISTAT package (Worsley et al., 2002) in MATLAB R2019a (Mathworks). The six motion parameters were included as covariates, as were time series from white matter and cerebrospinal fluid regions to account for nonspecific global fluctuations, and three cubic spline temporal trends.

The T1-weighted anatomical images were warped to MNI space using unified segmentation in SPM5 (Ashburner & Friston, 2005). Functional images were coregistered with structural images via coplanar T2-weighted structural images using SPM, and warped to MNI space.

Contrasts were created to compare each of the four conditions to the implicit Rest baseline. *Linguistic demand* was modeled with the contrast Semantic Difficult − Semantic Easy. Although this contrast captures linguistic demand only in the specific context of a lexical-semantic task, it is noteworthy that this contrast has previously been demonstrated to robustly activate core language regions in general, not just lexical-semantic regions (Wilson et al., 2018), supporting its use as a proxy for linguistic demand in general. *Non-linguistic demand* was modeled with the contrast Perceptual Difficult − Perceptual Easy. Again this is a specific instantiation of non-linguistic demand, but the striking similarity between regions modulated by non-linguistic demand across a range of diverse cognitive tasks (Fedorenko et al., 2013) suggests that any kind of non-linguistic demand can effectively serve as a proxy for non-linguistic demand in general. Language regions were identified with the contrast (Semantic Easy + Semantic Difficult) − (Perceptual Easy + Perceptual Difficult). Finally, the interaction of domain by difficulty was modeled by the contrast (Semantic Difficult − Semantic Easy) − (Perceptual Difficult − Perceptual Easy). Second level random effects analyses were performed and a cluster-defining threshold of *p* < 0.005 was applied. Correction for multiple comparisons (*p* < 0.05) was carried out based on permutation testing of the maximum cluster extent with the FSL tool *randomise* (Winkler et al., 2014). Specifically, null distributions were created by randomly inverting the signs of individual contrast images.

A region of interest (ROI) analysis was carried out to examine responses to each of the four active conditions in the MD network and the language network, using ROIs that were functionally defined in individual participants (Fedorenko et al., 2010). MD ROIs were defined based on the symmetrical image of the MD network described by Fedorenko et al. (2013) and available for download at http://imaging.mrc-cbu.cam.ac.uk/imaging/MDsystem. We used this image rather than our own data in order to avoid circularity, since we intended to test whether ROIs were modulated by perceptual demand, which is the same contrast we would use to define the MD network. We defined spheres of radius 8 mm around prominent peaks in each hemisphere. Twelve spheres were defined in the bilateral inferior frontal junction, anterior insula, pre-SMA/anterior-mid cingulate, dorsal premotor cortex, intraparietal sulcus, and occipito-temporal cortex. For the pre-SMA/anterior-mid cingulate ROI, we used peaks from sagittal slices *x* = ±6, because this region had only a single peak centered on the midline. Then, for each of the two runs, we defined individual ROIs as the top 10% of voxels within each sphere that had the highest *t* statistics for modulation by non-linguistic demand in the *other* run, and that were not modulated by language (uncorrected *p* > 0.1).

Language ROIs were based on the language regions identified in our own data. This did not introduce circularity, since the contrast to identify language regions was orthogonal to the contrasts under investigation in the ROI analysis. We further smoothed the language contrast image using a Gaussian filter with FWHM = 8 mm in order to identify maximally general peaks, then defined spheres of radius 8 mm around seven prominent peaks in the left hemisphere: the dorsal pars opercularis of the inferior frontal gyrus (IFG), pars triangularis of the IFG, SMA, fusiform gyrus, posterior superior temporal sulcus (STS), anterior STS, and hippocampus. Three additional spheres were also defined around prominent right hemisphere peaks that did not reach statistical significance but were clearly homotopic to left hemisphere language areas: the pars triangularis of the IFG, posterior STS, and anterior STS. Then, for each of the two runs, we defined individual ROIs as the top 10% of voxels within each sphere that had the highest *t* statistics for the language contrast in the *other* run, and that were not modulated by non-linguistic demand (uncorrected *p* > 0.1).

We used omnibus repeated measures ANOVAs in JMP and a series of *t* tests to determine which regions were modulated by linguistic demand, which regions were modulated by perceptual demand, and which regions showed interactions of domain by difficulty, and to compare the magnitude of these effects between the regions comprising each network. To account for multiple comparisons, *p* values for all of these tests were obtained from permutation testing using a custom procedure implemented in MATLAB. Specifically, null distributions for the effects of linguistic and non-linguistic demand and the interaction of domain by difficulty were created by randomly inverting the signs of individual contrast images, while null distributions for differential effects between regions were created by randomly permuting contrast estimates between regions within participants.

## RESULTS

Our study is concerned with cortical networks, so only cortical activations will be described in the text. Subcortical and cerebellar findings can be observed in the figures and tables.

### Behavioral Data

For accuracy, a repeated measures ANOVA with two within-subjects factors (domain, difficulty) revealed a main effect of difficulty with difficult conditions less accurate than easy conditions, *F*(1, 19) = 173.01, *p* < 0.0001, but no main effect of domain, *F*(1, 19) = 0.53, *p* = 0.48, and no interaction of domain by difficulty, *F*(1, 19) = 2.22, *p* = 0.15 (Figure 1B).

For reaction time, a repeated measures ANOVA revealed a main effect of difficulty with slower responses to difficult conditions compared to easy conditions, *F*(1, 19) = 151.13, *p* < 0.0001, but no main effect of domain, *F*(1, 19) = 4.21, *p* = 0.054, and no interaction of domain by difficulty, *F*(1, 19) = 1.88, *p* = 0.19 (Figure 1C).

The absence of significant domain by difficulty interactions for accuracy or reaction time is important, because it means that we can compare the neural correlates of linguistic demand and non-linguistic demand without this comparison being confounded by accuracy or reaction time. The lack of main effects of domain was welcome, though less important, since only the contrast used to identify language regions could be impacted by main effects of domain; for that reason, we were not concerned that the main effect of domain approached significance for reaction time.

One respect in which the domains did differ was in the nature of the errors made on the difficult conditions. On the Semantic Difficult condition, the total error rate of 18.0 ± 7.1% (*SD*) was made up of 14.4 ± 6.6% misses and 3.6 ± 3.2% false alarms, while on the Perceptual Difficult condition, the total error rate of 18.7 ± 6.5% was made up of 3.5 ± 1.9% misses and 15.2 ± 6.7% false alarms. To ensure that our imaging findings were not impacted by the differing ratios of “go” and “no-go” responses across conditions, all analyses described in the following sections were repeated with the inclusion of an additional explanatory variable modeling “go” versus “no-go” responses within blocks. This variable picked up left-hand button presses as expected, but its inclusion did not meaningfully change the results for any contrasts of interest.

### Brain Regions Modulated by Linguistic Demand

**Figure 2.**
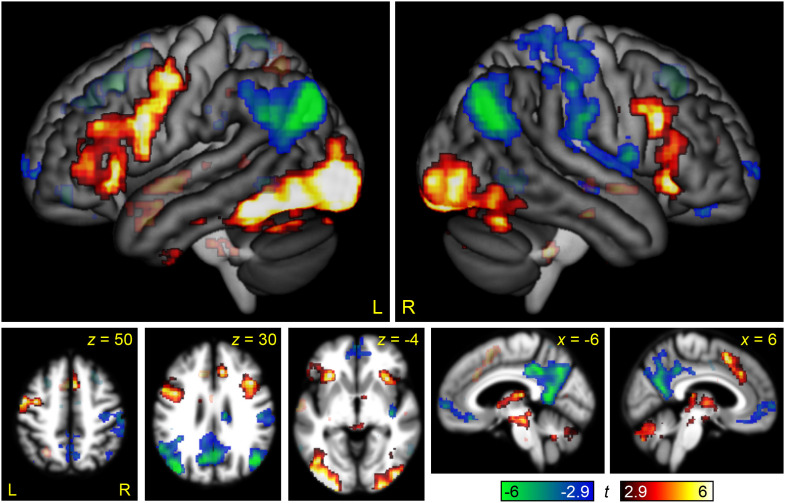
Brain regions modulated by linguistic demand. The contrast between the Semantic Difficult and Semantic Easy conditions is shown in hot colors, while the reverse contrast is shown in cool colors. Opaque = statistically significant, corrected for multiple comparisons; transparent = voxelwise *p* < 0.005, but did not meet cluster extent threshold.

The contrast between the Semantic Difficult and Semantic Easy conditions was used to identify brain regions modulated by linguistic demand (Figure 2, Table 2). In the left hemisphere, the regions that were differentially active for the more difficult condition included the left IFG (pars opercularis and triangularis) and sulcus, the inferior frontal junction, the precentral gyrus, the anterior insula, and an extensive occipito-temporal region extending anteriorly along the fusiform gyrus almost to the temporal pole; also notable was activation of the left pre-SMA/anterior-mid cingulate that did not meet the cluster extent threshold (*p* = 0.082). Activations in the right hemisphere were similar but less extensive: The inferior frontal activation was largely restricted to the ascending ramus of the Sylvian fissure, the inferior frontal junction, and the anterior insula, while the occipito-temporal region did not extend nearly as anteriorly; the right pre-SMA/anterior-mid cingulate did, however, reach significance. The regions that were deactivated by this contrast were bilateral and reflected the default mode network: the angular gyrus, precuneus, posterior cingulate, and ventromedial prefrontal cortex.

**Table 2.** Coordinates of activated regions

Brain region(s)	Extent (mm^3^)	Max *t*	MNI coordinates			*p*
			*x*	*y*	*z*	
*Linguistic demand*						
Left fusiform gyrus, inferior temporal gyrus, lingual gyrus, inferior and middle occipital lobe	33,432	13.61	−37	−71	−12	0.0017
Left IFG pars opercularis and triangularis, inferior frontal sulcus, inferior frontal junction, precentral gyrus, anterior insula	30,224	7.20	−43	15	20	0.0028
Right inferior occipital lobe	17,824	6.51	36	−78	−11	0.0085
Bilateral thalamus, basal ganglia, midbrain	13,928	7.44	−3	−12	1	0.013
Right IFG ascending ramus of the Sylvian fissure, inferior frontal junction, anterior insula	12,008	8.10	41	19	17	0.017
Bilateral cerebellum	6,792	6.44	3	−71	−28	0.036
Right pre-SMA/anterior-mid cingulate	5,680	6.20	9	24	38	0.046
*Negative linguistic demand*						
Bilateral precuneus, posterior cingulate	37,016	9.86	−1	−53	29	0.0010
Left angular gyrus	16,616	10.46	−45	−65	30	0.0091
Right supramarginal gyrus, postcentral gyrus	15,408	5.21	48	−27	44	0.011
Right angular gyrus	8,672	8.24	47	−66	31	0.027
Bilateral ventromedial prefrontal cortex	7,280	5.64	2	49	−6	0.036
Right posterior insula	5,712	5.84	41	−6	5	0.049
*Interaction of domain by difficulty*						
Left IFG pars triangularis and orbitalis, superior temporal gyrus, STS, middle temporal gyrus, hippocampus	45,112	7.22	−47	−4	−9	0.0013
Bilateral ventromedial prefrontal cortex, superior frontal gyrus	37,008	8.61	−1	50	18	0.0020
Bilateral precuneus, posterior cingulate	26,840	7.70	−1	−51	28	0.0038
Right superior temporal gyrus, STS, middle temporal gyrus	15,584	6.84	60	−14	−12	0.0099
*Negative interaction of domain by difficulty*						
Much of the bilateral multiple demand network, cerebellum	232,624	10.66	7	−53	16	< 0.0001
Left dorsal precentral gyrus and sulcus, superior frontal sulcus, pre-SMA, anterior-mid cingulate	14,776	7.47	−20	−1	54	0.012
Left anterior insula, basal ganglia	7,672	6.84	−28	18	8	0.029
Right thalamus	7,584	7.21	16	−15	11	0.029

*Note*. Coordinates are centers of mass.

### Voxelwise Comparisons Between Regions Modulated by Linguistic Demand, the MD Network, and the Language Network

We carried out several different analyses to investigate how the brain regions modulated by linguistic demand relate to the MD network and the language network.

**Figure 3.**
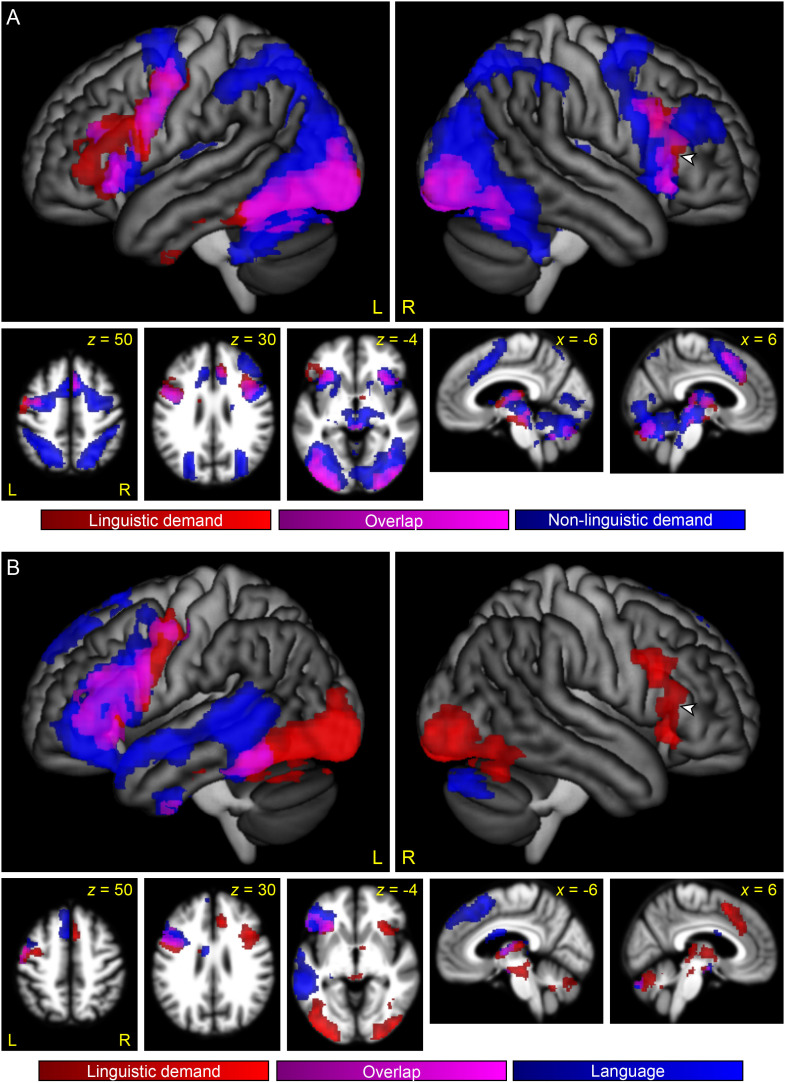
The relationship between regions modulated by linguistic demand, the multiple demand network, and the language network. (A) Regions modulated by linguistic demand are shown in red or violet. Regions modulated by non-linguistic demand, as revealed by the contrast between the Perceptual Difficult and the Perceptual Easy conditions, are shown in blue or violet. Violet indicates overlap. (B) Regions modulated by linguistic demand are shown in red or violet. Language regions, as revealed by the contrast of both semantic conditions to both perceptual conditions, are shown in blue or violet. Violet indicates overlap. Arrowheads indicate right IFG region that was modulated by linguistic demand, but was neither an MD region nor a language region.

**Figure 4.**
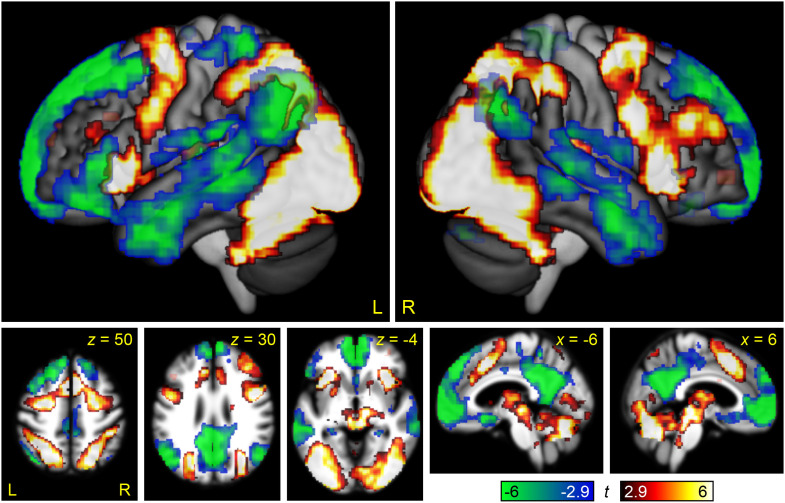
Brain regions modulated by non-linguistic demand. The contrast between the Perceptual Difficult and Perceptual Easy conditions is shown in hot colors, while the reverse contrast is shown in cool colors. Opaque = statistically significant, corrected for multiple comparisons; transparent = voxelwise *p* < 0.005, but did not meet cluster extent threshold.

First, we plotted the overlap between linguistic demand regions and MD regions (Figure 3A). MD regions were identified by the contrast between the Perceptual Difficult and Perceptual Easy conditions, which activated a widespread bilateral network (Figure 4), consistent with prior research.

We found that many of the regions that were modulated by linguistic demand belonged to the MD network, specifically, the bilateral inferior frontal junction extending especially in the left hemisphere onto the adjacent precentral gyrus, the bilateral anterior insula, the ventrolateral component of occipito-temporal regions bilaterally, and the right pre-SMA/anterior-mid cingulate. (Note that the left pre-SMA/anterior-mid cingulate narrowly missed significance for modulation by linguistic demand, as seen in Figure 2).

**Figure 5.**
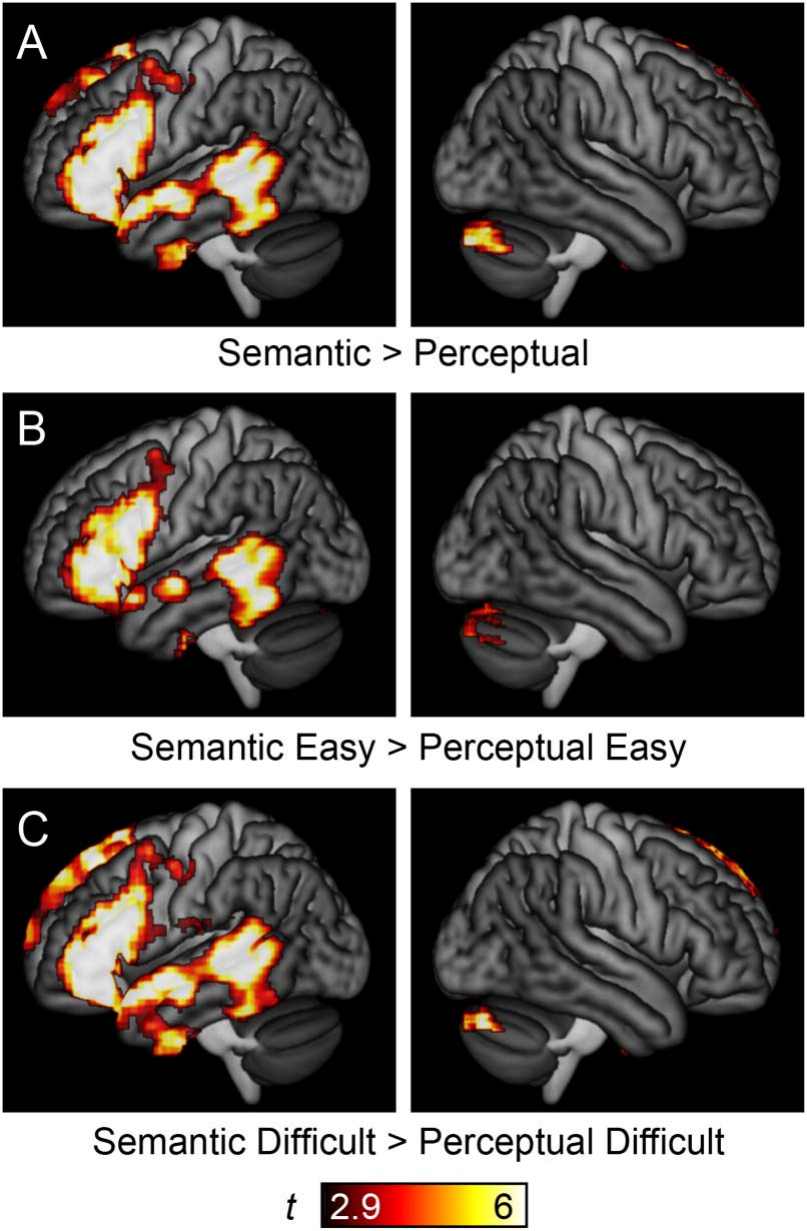
Language regions of the brain. (A) Language areas, as revealed by the contrast of (Semantic Easy + Semantic Difficult) − (Perceptual Easy + Perceptual Difficult). Note that contrasts between (B) the two easy conditions or (C) the two difficult conditions yielded similar results.

Next, we plotted the overlap between linguistic demand regions and language regions (Figure 3B). Language regions were identified by the contrast of both semantic matching conditions to both perceptual matching conditions, which activated strongly left-lateralized frontal and temporal language regions (Figure 5), consistent with prior research.

This analysis showed that several of the regions that were modulated by linguistic demand were language regions, specifically, left frontal regions (the pars opercularis and pars triangularis of the IFG, inferior frontal sulcus, dorsal precentral gyrus, and anterior insula) and a region extending anteriorly from the visual word form area along the length of the fusiform gyrus.

The largest cluster that was modulated by linguistic demand but was neither an MD region nor a left hemisphere language region was located in the right IFG, centered on the ascending ramus of the Sylvian fissure, which separates the pars opercularis from the pars triangularis (cluster extent = 1,456 mm^3^, maximum *t* = 4.67; center of mass MNI coordinates = 52, 26, 10, see arrowheads in Figure 3).

**Figure 6.**
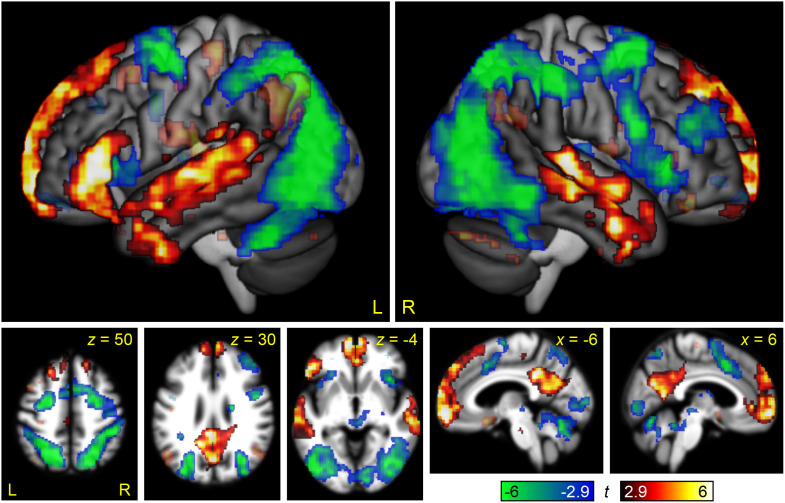
The interaction between linguistic demand and domain-general demand. Regions where modulation by linguistic demand was greater than modulation by domain-general demand are shown in hot colors, while the reverse contrast is shown in cool colors. Opaque = statistically significant, corrected for multiple comparisons; transparent = voxelwise *p* < 0.005, but did not meet cluster extent threshold.

Finally, we computed a whole brain interaction contrast of domain by difficulty, in order to directly compare modulation by linguistic and non-linguistic demand (Figure 6, Table 2). This interaction map revealed large scale differences between linguistic demand and non-linguistic demand. Core language regions in the left IFG (pars triangularis and orbitalis) and left superior temporal gyrus, STS, and middle temporal gyrus were differentially modulated by linguistic demand relative to non-linguistic demand, as were right hemisphere homotopic regions in the temporal lobe, and midline semantic/default mode regions in the anterior cingulate, medial prefrontal cortex, and the precuneus. Conversely, almost the entire MD network was differentially modulated by non-linguistic demand. It is important to note that many of these significant interactions were the consequence not of positive modulation by linguistic demand, but of negative modulation by non-linguistic demand, especially throughout the default mode network.

### Functionally Defined Regions of Interest

**Figure 7.**
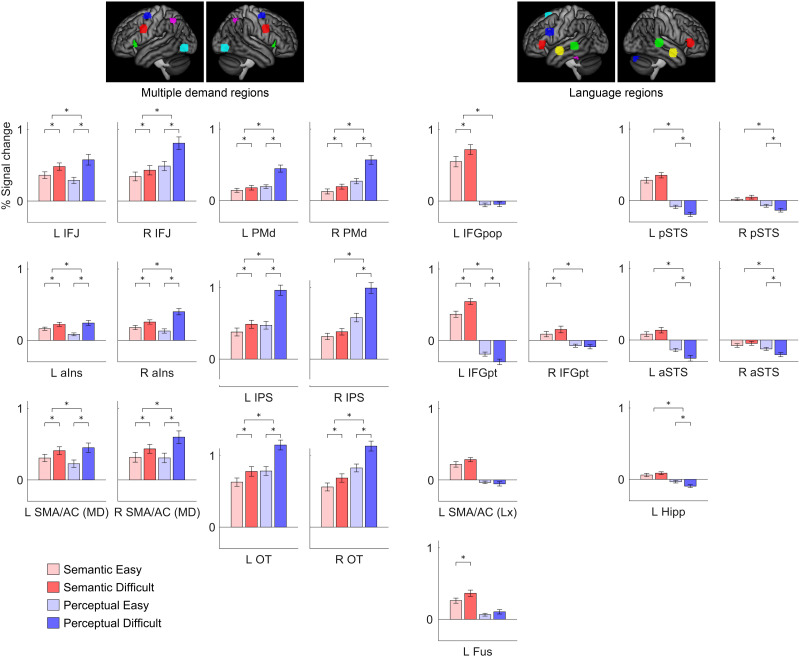
Functional region of interest analysis. Signal change for the four conditions in multiple demand regions, language regions, and right hemisphere regions homotopic to language regions. Error bars show standard error of the mean. The upper significance markers indicate the significance of the interaction of domain by difficulty. L = left; R = right; IFJ = inferior frontal junction; aIns = anterior insula; SMA/AC = pre-supplementary motor area/anterior-mid cingulate; PMd = dorsal premotor cortex; IPS = intraparietal sulcus; OT = occipitotemporal cortex; IFGpop = inferior frontal gyrus, pars opercularis; IFGpt = inferior frontal gyrus, pars triangularis; Fus = fusiform gyrus; pSTS = posterior superior temporal sulcus; aSTS = anterior superior temporal suclus; Hipp = hippocampus.

A limitation of voxelwise group analyses is that participants are aligned anatomically but not functionally, potentially resulting in the conflation of adjacent but functionally distinct brain regions (I. A. Blank et al., 2017; Fedorenko et al., 2010). Therefore, we next carried out a ROI analysis in which we plotted signal change as a function of domain (linguistic, non-linguistic) and difficulty (easy, difficult) in MD network and language network nodes that were functionally defined in individual participants (Figure 7, Table 3).

**Table 3.** Region of interest analysis

	Modulation by linguistic demand				Modulation by non-linguistic demand				Interaction of domain by difficulty			
	*d_z_*	*p*_unc_	*p*		*d_z_*	*p*_unc_	*p*		*d_z_*	*p*_unc_	*p*	
*MD network*												
L IFJ	1.37	< 0.0001	0.0002	*	1.25	< 0.0001	0.0002	*	−0.72	0.0044	0.018	*<
R IFJ	1.08	0.0001	0.0012	*	1.86	< 0.0001	< 0.0001	*	−1.43	< 0.0001	< 0.0001	*<
L aIns	0.77	0.0027	0.016	*	1.27	< 0.0001	0.0002	*	−0.98	0.0003	0.0022	*<
R aIns	0.95	0.0004	0.0033	*	1.89	< 0.0001	< 0.0001	*	−1.39	< 0.0001	< 0.0001	*<
L SMA/AC	0.94	0.0005	0.0034	*	1.83	< 0.0001	< 0.0001	*	−1.02	0.0002	0.0015	*<
R SMA/AC	1.18	< 0.0001	0.0007	*	1.81	< 0.0001	< 0.0001	*	−0.94	0.0005	0.0029	*<
L PMd	0.64	0.0098	0.048	*	1.65	< 0.0001	< 0.0001	*	−1.52	< 0.0001	< 0.0001	*<
R PMd	0.71	0.005	0.026	*	1.78	< 0.0001	< 0.0001	*	−1.59	< 0.0001	< 0.0001	*<
L IPS	1.02	0.0002	0.0019	*	2.29	< 0.0001	< 0.0001	*	−1.96	< 0.0001	< 0.0001	*<
R IPS	0.60	0.015	0.071		2.31	< 0.0001	< 0.0001	*	−1.82	< 0.0001	< 0.0001	*<
L OT	1.33	< 0.0001	0.0002	*	2.29	< 0.0001	< 0.0001	*	−1.57	< 0.0001	< 0.0001	*<
R OT	1.07	0.0001	0.0014	*	1.83	< 0.0001	< 0.0001	*	−1.31	< 0.0001	0.0002	*<
*Language network*												
L IFGpop	1.06	0.0001	0.001	*	0.09	0.68	1.00		0.73	0.004	0.025	*
L IFGpt	1.20	< 0.0001	0.0002	*	−1.13	0.0001	0.0007	*<	1.63	< 0.0001	< 0.0001	*
L SMA/AC	0.51	0.033	0.18		−0.22	0.34	0.96		0.57	0.020	0.11	
L Fus	1.15	0.0001	0.0002	*	0.49	0.041	0.27		0.57	0.019	0.10	
L pSTS	0.42	0.077	0.35		−1.12	0.0001	0.0009	*<	1.07	0.0001	0.0017	*
L aSTS	0.49	0.042	0.22		−0.98	0.0003	0.0026	*<	0.84	0.0014	0.011	*
L Hipp	0.38	0.10	0.44		−0.80	0.0019	0.016	*<	0.75	0.0034	0.022	*
R IFGpt	0.73	0.0042	0.032	*	−0.19	0.40	0.98		0.69	0.0058	0.037	*
R pSTS	0.39	0.097	0.42		−0.91	0.0006	0.0051	*<	0.93	0.0005	0.005	*
R aSTS	0.45	0.057	0.27		−1.39	< 0.0001	< 0.0001	*<	1.16	0.0001	0.0007	*

*Note*. *p*_unc_ = uncorrected *p* value; * = statistically significant after correction for multiple comparisons; < = negative modulation; see Figure 7 caption for abbreviations.

All twelve MD regions examined showed similar functional profiles. All twelve were modulated by linguistic demand (except for one region with *p* = 0.07), reflecting greater sensitivity of functional ROI-based analysis compared to the voxelwise analysis. All twelve regions were modulated by non-linguistic demand, and all twelve showed interactions whereby modulation was greater by non-linguistic demand than linguistic demand; these findings were consistent with the voxelwise analyses. Although all twelve MD regions were modulated by linguistic demand, they differed in the extent to which they were modulated, *F*(4.85, 92.23) = 5.37, *p* = 0.0003 (Supplementary Table 1; supporting information can be found online at https://doi.org/10.1162/nol_a_00031). The left inferior frontal junction and left occipito-temporal region were most strongly modulated, while left dorsal premotor cortex was least strongly modulated. The twelve regions were also not equivalent in their relative modulation by linguistic and non-linguistic demand, *F*(5.06, 96.18) = 14.49, *p* < 0.0001 (Supplementary Table 2). The left anterior insula showed the most similar modulation across domains, while the bilateral intraparietal sulci and dorsal premotor cortex showed the greatest discrepancy between domains. Of note, no homotopic pairs of regions differed from one another in terms of modulation by linguistic demand or the interaction of domain by difficulty, though the anterior insula showed a trend toward an interaction, with less of a discrepancy between domains in the left anterior insula compared to the right (*p* = 0.058).

The ten language regions (including homotopic regions) examined showed a more diverse array of functional profiles (Figure 7, Table 3). All language regions were numerically modulated by linguistic demand, though this modulation was statistically significant only for the left IFG pars opercularis, left IFG pars triangularis, left fusiform gyrus, and right IFG pars triangularis, consistent with the voxelwise analysis described in the previous section. The differences between regions in extent of modulation were significant, *F*(3.68, 69.90) = 8.62, *p* < 0.0001 (Supplementary Table 3), and were driven mainly by the left IFG pars opercularis and left IFG pars triangularis, which were more strongly modulated than almost every other region. None of the language regions were modulated by non-linguistic demand, and several of them were negatively modulated: the left IFG pars triangularis, left posterior STS, left anterior STS, left hippocampus, and right posterior STS. Eight of the ten language regions showed significant interactions of domain by difficulty: the left IFG pars opercularis, left IFG pars triangularis, left posterior STS, left anterior STS, left hippocampus, right IFG pars triangularis, right posterior STS, and right anterior STS, and there were differences between regions in their relative modulation by linguistic and non-linguistic demand, *F*(4.25, 80.74) = 7.05, *p* < 0.0001 (Supplementary Table 4). Specifically, the left IFG pars triangularis showed a greater discrepancy between domains than almost every other region. Among the homotopic pairs of nodes examined, the left IFG pars triangularis showed greater modulation by linguistic demand than the right IFG pars triangularis (*p* = 0.034) as well as a greater discrepancy of modulation relative to non-linguistic demand (*p* = 0.0001). There were no interhemispheric differences in the temporal lobes.

## DISCUSSION

In many diverse cognitive paradigms, task difficulty modulates the MD network in a highly stereotyped manner (Duncan & Owen, 2000; Fedorenko et al., 2013). Our results indicate that the neural correlates of linguistic demand are strikingly different. We found that linguistic demand modulated the MD network to a lesser extent than non-linguistic demand, and that this discrepancy was more pronounced for some nodes than others. Outside of the MD network, linguistic demand modulated a subset of language regions, with the most robust modulation in the left IFG; a homotopic region in the right IFG was also modulated. Finally, if linguistic demand is conceptualized relative to non-linguistic demand, linguistic demand could be seen to modulate left temporal language regions as well as a wider bilateral semantic network.

### How MD Regions Respond to Linguistic Demand

All twelve MD regions examined were modulated by linguistic demand, but all twelve were less modulated by linguistic demand than they were by non-linguistic demand, suggesting that task difficulty in a linguistic context depends less on the MD network than task difficulty in non-linguistic contexts. This may reflect the fact that linguistic demand draws also on brain regions outside the MD network, as discussed below. The discrepancy between linguistic and non-linguistic demand was particularly pronounced in the dorsal premotor and intraparietal sulcus nodes, which belong to the dorsal attention network and are involved in spatial attention and eye movements (Corbetta & Shulman, 2002, 2011).

A number of previous studies have shown that manipulations of various kinds of linguistic difficulty modulate likely MD regions (Binder et al., 2004; Binder et al., 2005; Blumstein et al., 2005; Eckert et al., 2009; Erb et al., 2013; Ihnen et al., 2015; Mollica et al., 2020; Noppeney & Price, 2004; Piai et al., 2013; Sabsevitz et al., 2005; Vaden et al., 2013; Wilson et al., 2016). What is novel about our study is that it is the first, to our knowledge, to match task structure, accuracy, and reaction time across linguistic and non-linguistic tasks so as to permit direct statistical comparisons between domains.

There was no indication that recruitment of the MD network for increasing linguistic demand was left-lateralized. This contrasts with recruitment of the MD network for language tasks relative to resting baselines, which is modestly left-lateralized (Diachek et al., 2020). Taken together, these two findings suggest that the basic demands of performing a language task draw somewhat more on left hemisphere MD regions, but that additional demands as the language task becomes more difficult recruit MD regions in both hemispheres similarly.

It is unclear to what extent MD regions support language processing in real world language use, as opposed to only in metalinguistic tasks. I. A. Blank and Fedorenko (2017) examined intersubject correlations between participants listening to the same narratives in MD regions and language regions. They found stronger intersubject correlations in language regions than MD regions, and focused their interpretation on this difference, but it is noteworthy that intersubject correlations throughout the MD network were still highly significant. We interpret this as evidence for modulation of the MD network by the systematic time-varying demands of (relatively) ecologically valid language comprehension. However other studies from the same group have provided evidence against MD involvement in real world language processing, including lack of MD recruitment in the absence of overt tasks (Diachek et al., 2020), lack of MD modulation by surprisal (Shain et al., 2020), and lack of MD modulation by online measures of incremental processing load (Wehbe et al., 2020). This question is not central to our study, because we are primarily concerned with informing the interpretation of studies of language processing in aphasia, which have usually involved metalinguistic tasks. But going forward, the resolution of this question will be important, because we are interested in understanding the neural mechanisms of real world language processing, not just performance of language tasks.

### How Language Regions Respond to Linguistic Demand

Left frontal language regions—the pars triangularis and pars opercularis of the IFG—were strongly modulated by linguistic demand, consistent with many previous findings (Binder et al., 2005; Bornkessel et al., 2005; Graves et al., 2007; Graves et al., 2010; Just et al., 1996; Makuuchi et al., 2009; Mason et al., 2003; Obleser et al., 2011; Rodd et al., 2005; Roskies et al., 2001; Sabsevitz et al., 2005; Stromswold et al., 1996; Wilson et al., 2016). Ventral temporal language regions extending along the fusiform gyrus were also modulated by linguistic demand.

In contrast, the core language regions of the lateral temporal lobe presented a more complex picture. These regions were not modulated by linguistic demand in either the whole brain analysis or the ROI analysis. Several previous studies employing semantic tasks have similarly shown no modulation of temporal lobe regions by task difficulty (Binder et al., 2005; Noppeney & Price, 2004; Sabsevitz et al., 2005; but cf. Badre et al., 2005 for a positive finding). One possible interpretation is that these regions are recruited for semantic processing in an “all or none” manner (Sabsevitz et al., 2005). However, our preferred interpretation builds on the observation that temporal lobe regions are involved in conceptual processing even in the resting state (Binder et al., 1999). We propose that linguistic demand as manipulated in the present study may shift the relative balance of introspective versus externally cued semantic processing, without greatly affecting the overall extent of such processing (see also McKiernan et al., 2003).

Importantly, left lateral temporal language regions were deactivated by perceptual processing relative to rest, and were deactivated further still in the Difficult Perceptual condition. These patterns can be interpreted as reflecting the inhibition of conceptual processing by an attention-demanding task (Binder et al., 1999; McKiernan et al., 2003; Shulman et al., 1997). The lack of modulation by linguistic demand in combination with differential deactivation by perceptual demand yielded significant domain by difficulty interactions. If linguistic demand is conceptualized as a relative concept, with non-linguistic demand as a point of reference, then we can argue that these left temporal lobe language regions are in fact modulated by linguistic demand, but that this is masked in the simple linguistic demand contrast because of the simultaneous decrease in introspective conceptual processing that occurs as a general function of increasing task difficulty.

### How Regions Outside the MD and Language Networks Respond to Linguistic Demand

Most regions modulated by linguistic demand belonged to either the MD network or the language network. In the voxelwise analysis, the largest cluster meeting neither of these criteria was localized to the right IFG, between the pars opercularis and pars triangularis. This region was homotopic to a left hemisphere language region, and was surrounded by MD regions in the anterior insula, inferior frontal junction, and middle frontal gyrus, but was not itself modulated by non-linguistic demand. The modulation of the right IFG by linguistic demand but not by perceptual demand was confirmed in the ROI analysis.

This positive finding of right hemisphere recruitment as language processing becomes more demanding stands in contrast to two previous studies that sought to identify right hemisphere recruitment for greater linguistic demand using functional transcranial Doppler sonography (Dräger & Knecht, 2002) and fMRI (Dräger et al., 2004); however, it is possible that the more difficult condition in those studies did not result in a greater extent of language processing, since a word completion task was used, and participants generated more words in the easier condition. Another recent study found that the IFG maintained a lateralized activation pattern as the difficulty of a verbal working memory task increased, unlike other prefrontal regions in which activations became more bilateral with increasing task difficulty (Höller-Wallscheid et al., 2017). However, in that study, the maintained lateralization pattern reflected modulation of both left and right IFG by task difficulty, consistent with our findings.

Right hemisphere temporal lobe regions, as well as the wider bilateral semantic network, which is closely related to the default mode network (Binder et al., 2009), showed a similar pattern to left temporal language regions. These regions were not modulated by linguistic demand, but they were negatively modulated by non-linguistic demand, and therefore significant domain by difficulty interactions were observed in whole brain and ROI analyses. Similar to left temporal regions, a relative conception of linguistic demand would imply that these regions actually are in a sense modulated by linguistic demand, in that they do not deactivate as a function of difficulty, as would be expected in a non-linguistic task.

### Challenges in Operationalizing Linguistic Demand

The most significant limitations of our study relate to the operationalization of linguistic demand. First and foremost, we investigated only one type of language task: a semantic decision task. Our rationale for choosing this task as a proxy for language processing in general was that it has previously been demonstrated to activate core language regions in a reliable and valid manner (Wilson et al., 2018). Some partial support for the choice of this task as a proxy for language processing in general comes from our finding that the language regions most strongly modulated by linguistic demand in this task were the pars triangularis and opercularis of the IFG, neither of which are generally considered semantic regions (Binder et al., 2009). But the language system has many subcomponents and there are many different kinds of linguistic demand (Deschamps & Tremblay, 2014; Graves et al., 2010; McGettigan et al., 2011; Wilson et al., 2009). Some linguistic processes depend on regions outside the core language network identified by the semantic decision task, such as phonological encoding, which depends on the left supramarginal gyrus (Price et al., 1997; Yen et al., 2019). Moreover, even language domains that depend predominantly on the core regions identified by our task may differ from our task in terms of modulation by demand. For example, a number of studies that have shown that syntactic complexity modulates not just inferior frontal cortex (consistent with our findings) but also the posterior temporal language region (which was not significantly modulated in the present study) (I. Blank et al., 2016; Wilson et al., 2010).

Second, the Semantic Easy and Semantic Difficult conditions differ not just in the demands they make on the linguistic system, but also in the demands they make on the conceptual semantic system. This is relevant to interpreting the domain by difficulty interaction effects we observed throughout the bilateral semantic network, which are more likely to reflect modulation by “semantic demand” rather than linguistic demand per se.

Third, the linguistic and non-linguistic conditions differ in ways other than the presence versus absence of linguistic content. One is a task of conceptual matching, the other a task of judging perceptual identity. Experientially, the semantic task involves retrieving meanings for each word, and then searching for any possible connection between them. In contrast, the perceptual identity task entails looking back and forth between the two stimuli, searching for differences. In the semantic task, a successful search leads to a button press, while in the perceptual task, a successful search (i.e., the identification of a difference between the symbol strings) leads to the withholding of a button press. These structural differences between the tasks explain why most of the errors in the Semantic Difficult condition were misses, while most of the errors in the Perceptual Difficult condition were false alarms. While an additional explanatory variable accounting for the difference between “go” and “no-go” trials did not substantially change any of our findings, it is nevertheless a limitation that the tasks, while structured similarly, entailed somewhat different strategies.

Finally, our study was focused on modulation by demand, and not on initial recruitment of MD regions or other regions in the easy conditions. There were potentially informative differences between the linguistic and non-linguistic conditions even on the easy conditions. For example, the left inferior frontal junction showed more activity for the Semantic Easy condition than the Perceptual Easy condition, while the reverse was true of the right inferior frontal junction. It is possible that regions that were more strongly activated for the easy conditions may have had less potential to demonstrate further increases in activation for the difficult conditions.

### Implications for Studies of Neuroplasticity in Aphasia

Notwithstanding the limitations described in the previous section, our findings have clear implications for the interpretation of functional imaging studies of neuroplasticity in aphasia. Variables associated with task difficulty such as accuracy and reaction time have dramatic effects on functional signal (Binder et al., 2005; Fedorenko et al., 2013; Yarkoni et al., 2009), and task difficulty has been shown to modulate extent of activation in individuals with aphasia (Fridriksson & Morrow, 2005). Recently, Geranmayeh et al. (2014) suggested that many activations that have been attributed to reorganization of language processing may instead reflect effects of task difficulty. They speculated that such effects might be observed in several domain-general networks: the dorsal attention network, cingulo-opercular network, and fronto-parietal control network. Our findings—that there are major differences between the neural correlates of linguistic and non-linguistic demand—suggest some refinements to this argument. Our findings provide a more specific set of expectations about which brain regions might be expected to show activation due to task difficulty effects: Some MD regions are more strongly modulated by linguistic demand than others, and not just MD regions are modulated by linguistic demand, but also some language regions, especially left frontal regions, along with the homotopic region in the right IFG.

Geranmayeh et al. (2014) addressed several specific reported findings. They argued that the transient upregulation of the right anterior insula reported by Saur et al. (2006) might reflect increased activity related to task difficulty in a node of the cingulo-opercular network. Our findings confirm that the right insula is indeed robustly modulated by linguistic demand. In another case study, Geranmayeh et al. (2014) suggested that an atypical functional response profile in the right STS reported by Leff et al. (2002) might reflect engagement of a node of the ventral attention network. We found no evidence for modulation of the right STS or any nearby region by linguistic demand, suggesting that this finding is unlikely to reflect a task difficulty confound.

The right IFG, specifically the pars opercularis, is the brain region that has most often been reported to show increased activation in individuals with aphasia relative to matched control participants (S. C. Blank et al., 2003; Wilson & Schneck, 2021). The present finding that this region is modulated by linguistic demand suggests that this often-replicated finding may reflect the increased task difficulty experienced by individuals with aphasia.

We concur with Geranmayeh et al. (2014) in advocating caution in the interpretation of findings localized to MD regions, or in other regions that are modulated by linguistic demand, but it is also important to emphasize that such activations are not necessarily artifactual. It is plausible that the neural changes that support recovery from aphasia may include upregulation or even repurposing of domain-general regions, and that this may represent genuine compensatory reorganization rather than simply task performance confounds (Brownsett et al., 2014; DeMarco et al., 2018; Geranmayeh et al., 2017). It may prove difficult to distinguish between these two interpretations.

In general, in studies of neuroplasticity in aphasia, the interpretation of activation differences between groups, or correlations with behavior or time, remains complex and challenging. In many cases, performance on the control conditions will also need to be considered, which will entail considering the nature of interactions of domain by difficulty, which as we have shown yield a somewhat different picture than linguistic demand alone.

A promising direction for future research will be to investigate the neural correlates of linguistic demand in individuals with aphasia (Fridriksson & Morrow, 2005). We studied only neurologically normal individuals; moreover, our participants were younger and more educated than the typical stroke population. The lesions that cause aphasia almost always impact the MD network as well, so it is an important open question how damage to various nodes of both networks impacts the recruitment of surviving brain regions as a function of linguistic demand.

## ACKNOWLEDGMENTS

We thank Michael de Riesthal, Evgeniia Diachek, Melissa Duff, Jillian Entrup, Evelina Fedorenko, Deborah Levy, Sarah Schneck, and Lucina Uddin for valuable discussions and input, Marisa Bush, Josh Hageman, Clair Jones, Leslie McIntosh, and Chris Thompson for assistance with acquisition of MRI data, two anonymous reviewers for constructive feedback, and the individuals who participated in the study. This research was supported in part by the National Institute on Deafness and Other Communication Disorders.

## FUNDING INFORMATION

Stephen M. Wilson, National Institute on Deafness and Other Communication Disorders (http://dx.doi.org/10.13039/100000055), Award ID: R01 DC013270. Stephen M. Wilson, National Institute on Deafness and Other Communication Disorders (http://dx.doi.org/10.13039/100000055), Award ID: R21 DC016080.

## AUTHOR CONTRIBUTIONS

**Ian A. Quillen**: Methodology; Investigation; Formal analysis; Visualization; Writing – original draft; Writing – review & editing. **Melodie Yen**: Methodology; Investigation; Writing – review & editing. **Stephen M. Wilson:** Conceptualization; Methodology; Formal analysis; Visualization; Software; Writing – original draft; Writing – review & editing; Funding acquisition.

## TECHNICAL TERMS

The MD network: A bilateral network including the inferior frontal junction, anterior insula, pre-supplementary motor area, anterior-mid cingulate, and intraparietal sulcus that supports cognitive flexibility in many diverse contexts.
Linguistic demand: Refers to the additional processing requirements of more difficult linguistic tasks relative to easier linguistic tasks.
Non-linguistic demand: Refers to the additional processing requirements of more difficult non-linguistic cognitive tasks relative to easier tasks.
