# Supplementary material for: Distinct Neural Correlates of Linguistic and Non-Linguistic Demand
Source: Neurobiol Lang (Camb). 2021 Mar 29;2(2):202–25. doi: 10.1162/nol_a_00031 (PMC8475781; doi:10.1162/nol_a_00031)
Supplement: Supplementary file 1 [file nol-2-2-202-s001.pdf]

Supplementary Tables

Supplementary Table 1. Modulation by linguistic demand: *p* values for pairwise comparisons between multiple demand regions

	L IFJ	R IFJ	L aIns	R aIns	L SMA	R SMA	L PMd	R PMd	L IPS	R IPS	L OT
L IFJ											
R IFJ	.85										
L aIns	.38	.98									
R aIns	.80	1	.88								
L SMA	1	1	.65	.95							
R SMA	1	.85	.18	.21	1						
L PMd	.016 *	.14	.79	.080	.10	.013 *					
R PMd	.78	.99	1	1	.98	.72	.88				
L IPS	1	.99	.68	.93	1	1	.044 *	.53			
R IPS	.77	1	1	1	.94	.69	.93	1	.19		
L OT	.99	.24	.059	.11	.43	.96	.0002 *	.11	.44	.0051 *	
R OT	1	.94	.5	.76	1	1	.032 *	.54	1	.11	.86

All *p* values are corrected for multiple comparisons. * = $p < .05$. See Figure 7 caption for abbreviations.

Supplementary Table 2. Modulation by linguistic demand relative to non-linguistic demand: p values for pairwise comparisons between multiple demand regions

	L IFJ	R IFJ	L aIns	R aIns	L SMA	R SMA	L PMd	R PMd	L IPS	R IPS	L OT
L IFJ											
R IFJ	.57										
L aIns	.97	.0063 *									
R aIns	1	.88	.058								
L SMA	1	.019 *	1	.082							
R SMA	1	.76	.46	1	.36						
L PMd	.98	1	.01 *	1	.067	.99					
R PMd	.89	1	.0035 *	.98	.0021 *	.80	1				
L IPS	.0006 *	.0004 *	<.0001 *	.0004 *	<.0001 *	.0026 *	.0002 *	.0076 *			
R IPS	.0001 *	.014 *	.0001 *	.018 *	<.0001 *	.0046 *	.0011 *	.019 *	.99		
L OT	.97	1	.024 *	1	.026 *	.99	1	1	.0001 *	.0003 *	
R OT	1	.98	.60	1	.61	1	1	.87	.0021 *	.0044 *	.95

All p values are corrected for multiple comparisons. * = $p < .05$. See Figure 7 caption for abbreviations.

Supplementary Table 3. Modulation by linguistic demand: *p* values for pairwise comparisons between language regions

	L IFGpop	L IFGpt	L SMA	L Fus	L pSTS	L aSTS	L Hipp	R IFGpt	R pSTS
L IFGpop									
L IFGpt	1								
L SMA	.011 *	.0005 *							
L Fus	.52	.34	.94						
L pSTS	.014 *	.0072 *	1	.99					
L aSTS	.034 *	.0065 *	1	.71	1				
L Hipp	.010 *	.0087 *	.97	.019 *	.99	.99			
R IFGpt	.14	.034 *	1	.87	1	1	.88		
R pSTS	.010 *	.0023 *	.91	.12	.97	.98	1	.63	
R aSTS	.019 *	.0062 *	.94	.13	.99	.99	1	.71	1

All *p* values are corrected for multiple comparisons. * = $p < .05$. See Figure 7 caption for abbreviations.

Supplementary Table 4. Modulation by linguistic demand relative to perceptual demand: *p* values for pairwise comparisons between language regions

	L IFGpop	L IFGpt	L SMA	L Fus	L pSTS	L aSTS	L Hipp	R IFGpt	R pSTS
L IFGpop									
L IFGpt	.013 *								
L SMA	.82	.0001 *							
L Fus	.61	.0001 *	1						
L pSTS	1	.025 *	.26	.11					
L aSTS	1	.40	.67	.47	1				
L Hipp	.97	.0079 *	1	1	.63	.89			
R IFGpt	.78	.0001 *	1	1	.14	.54	1		
R pSTS	.91	.0008 *	1	1	.33	.85	1	1	
R aSTS	.99	.0020 *	1	.79	.74	.89	.99	.92	1

All *p* values are corrected for multiple comparisons. * = $p < .05$. See Figure 7 caption for abbreviations.
